# Beyond CT: A Case Analysis of Serial [18F]FDG PET/CT for Assessment of Necrosis and Early Recurrence in Colorectal Liver Metastases

**DOI:** 10.7759/cureus.51393

**Published:** 2023-12-31

**Authors:** Akram Al-Ibraheem, Saad Ruzzeh, Mohannad Badarneh, Dhuha Al-Adhami, Ahmad Telfah

**Affiliations:** 1 Department of Nuclear Medicine and PET/CT, King Hussein Cancer Center (KHCC), Amman, JOR; 2 School of Medicine, University of Jordan, Amman, JOR; 3 Department of Medicine, King Hussein Cancer Center (KHCC), Amman, JOR

**Keywords:** indolent metastasis, colorectal liver metastasis, pet/ct, peripheral hypermetabolism, [18f]fdg, transient necrosis

## Abstract

Colorectal cancer is a common malignancy, with the liver being the most frequent site of metastases. [^18^F] Fluorodeoxyglucose ([^18^F]FDG) positron emission tomography/computed tomography (PET/CT) has emerged as a valuable tool in detecting and evaluating liver metastases and extrahepatic disease. Herein, we present a case of a 76-year-old male with colorectal cancer associated with lung and liver metastases. The patient received 12 chemoimmunotherapy cycles and was then put on maintenance cetuximab; serial [^18^F]FDG PET/CT scans were utilized to evaluate treatment response. The patient exhibited a positive response to chemoimmunotherapy, with regression of rectal disease and resolution of pulmonary metastatic nodules. Serial [^18^F]FDG PET/CT scans unveiled three distinct necrotic patterns. The case report advocates that [^18^F]FDG PET/CT plays an important role in evaluating colorectal liver metastases (CRLM) response to treatment, identifying transient necrosis, early recurrence, and emphasizing the limitations of post-treatment CT scans in identifying early CRLM recurrence. Integrating functional imaging, particularly [^18^F]FDG PET/CT, promises for management monitoring and surveillance of CRLM patients.

## Introduction

Colorectal cancer is the third most common cancer in both genders [1], with the liver being the most common metastatic site [2]. Advancements in imaging techniques have improved the ability to detect and accurately characterize focal liver lesions [3]. Positron emission tomography/computed tomography (PET/CT) combines the metabolic benefits of [^18^F] fluorodeoxyglucose ([^18^F]FDG) uptake with the anatomical information obtained from CT imaging [4]. PET/CT demonstrates commendable sensitivity and specificity for detecting, evaluating, and following patients after the treatment of colorectal liver metastases (CRLM), aiding in assessing treatment efficacy and detecting potential recurrence [5,6].

Treatment options for CRLM include surgery, chemotherapy, chemo/radioembolization, and percutaneous techniques like microwave and radiofrequency ablation [7]. Surgical resection is feasible in about 20% of cases, but a multidisciplinary approach involving oncologists, surgeons, and specialists is crucial for tailoring optimal treatment plans [8]. Patients with CRLM can access diverse systemic therapies, including chemotherapy, biologics, and emerging immunotherapy [9]. Systemic therapies can induce changes in the imaging characteristics and microscopic composition of treated liver metastases, which can carry prognostic significance and imaging dilemmas [2].

While numerous studies have defined the proportion of patients with CRLM who respond to chemotherapy, the duration of sustained changes after treatment completion remains unexplored. In a large proportion of these patients, the treatment effect is transient, and disease progression occurs rapidly after stopping therapy [10].

In this report, we present a case of a 76-year-old male with colorectal cancer and liver metastases. The patient received chemoimmunotherapy, and his response was evaluated and monitored using serial [^18^F]FDG PET/CT scans. This case study aims to shed light on the patterns of necrosis and early recurrence of CRLM post-chemotherapy that may be encountered in [^18^F]FDG PET/CT scans in CRLM.

## Case presentation

**Figure 1:**
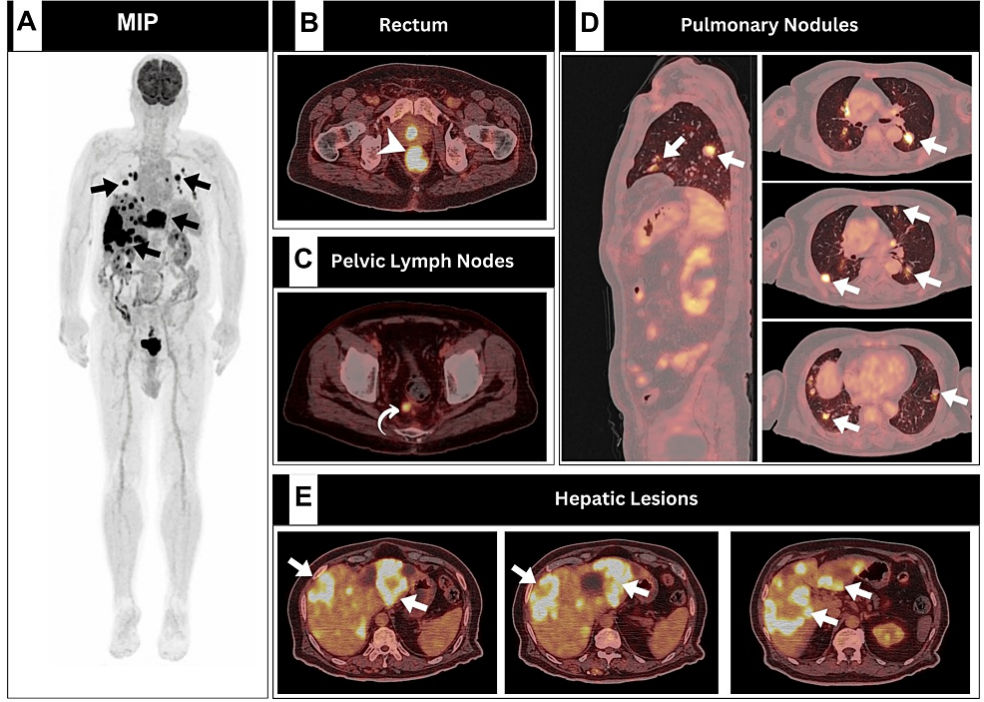
Baseline [18F]FDG PET/CT performed for the detection of unknown primary and subsequent staging A: Maximum intensity projection. B: Rectal transaxial PET/CT. C: Pelvic transaxial PET/CT. D: Sagittal and transaxial thoracic PET/CT. E: Hepatic transaxial PET/CT images revealed evidence of hypermetabolic primary rectal malignancy (arrowhead) associated with a few hypermetabolic perirectal lymph nodes (curved arrow) and multiple metastatic hepatic and pulmonary lesions (arrows). [^18^F]FDG PET/CT - [^18^F] fluorodeoxyglucose positron emission tomography/computed tomography

A 76-year-old male patient with a negative family history of malignancy presented with two months history of intermittent bleeding per rectum. Upon physical examination, he was found to have mild anemia (with hemoglobin of 10.6). In addition, serum carcinoembryonic antigen (CEA) and carbohydrate antigen 19-9 (CA-19-9) levels were found to be elevated, with values of 1200 and 249 ng/mL, respectively. Otherwise, all other laboratory tests were unremarkable. Abdominal magnetic resonance imaging (MRI) revealed multiple liver lesions suggestive of metastatic disease, prompting further evaluation. [^18^F]FDG PET/CT scan identified a hypermetabolic rectal mass, along with a few hypermetabolic perirectal lymph nodes, hypermetabolic metastatic liver lesions, and pulmonary nodules (Figure 1). Notably, the liver demonstrated multiple lesions of various density and metabolic activity (Figure 1E).

Histopathological examination confirmed moderately differentiated adenocarcinoma, leading to a stage IV rectal malignancy diagnosis. A multidisciplinary clinic (MDC) recommended a chemoimmunotherapy regimen consisting of folinic acid, fluorouracil, oxaliplatin (FOLFOX), and cetuximab immunotherapy.

**Figure 2:**
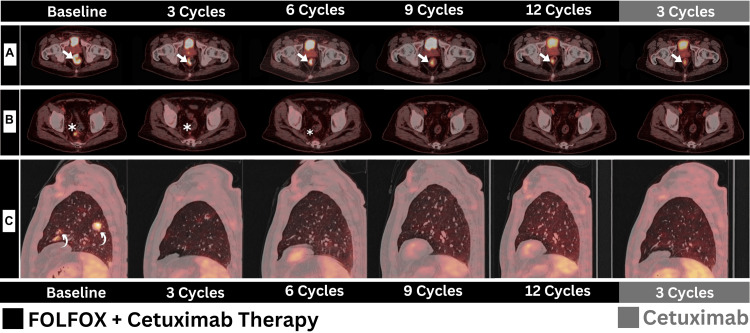
Serial fused PET/CT images captured from baseline and following each of the three cycles of therapy received A: A gradual regression in primary rectal malignancy was witnessed (arrows), reflecting both metabolic and morphologic responses on axial PET/CT images of the rectum. B: A similar response (asterisk) was also achieved for the few perirectal lymph nodes following the first six cycles, followed by a complete response. C: An earlier response was achieved for metastatic pulmonary nodules (visualized on sagittal PET/CT images), fading out after only three cycles of therapy (curved arrows) and maintaining complete resolution beyond that. FOLFOX - folinic acid, fluorouracil, oxaliplatin

Notably, radiotherapy was deemed unfeasible to address the systemic nature of the disease, particularly in the presence of lung metastasis (Figure 1D). After completing 12 cycles of chemoimmunotherapy, a total of three cycles of cetuximab-only therapy was offered as a maintenance regimen. Concomitantly, serial [^18^F]FDG PET/CT scans were employed following each and every three cycles of therapy for response assessment (Figure 2).

The primary rectal disease showed gradual improvement throughout the course of therapy (Figure 2A). Perirectal lymph nodes achieved partial response following the first three and six cycles and demonstrated complete metabolic response (CMR) afterwards (Figure 2B). An earlier response pattern was achieved for metastatic pulmonary nodules, achieving CMR only after six cycles (Figure 2C).

The main objective of conducting sequential [^18^F]FDG PET/CT scans was to observe the response patterns of CRLM lesions, with the added benefit of obtaining thorough anatomical and functional information (Table 1).

**Table 1:** Key differences in the advantages and disadvantages of computed tomography vs. positron emission tomography/computed tomography

Entity	PET/CT	CT
Insights provided	Functional, metabolic, and gross anatomical details	Anatomical and morphological
Availability	Limited availability	Widely available
Anatomic details	Limited spatial resolution	High spatial resolution for detailed anatomy
Whole-body Imaging	Allows whole-body assessment	Primarily focuses on the scanned region
Detection of early recurrence	Capable	Limited
Differentiation of necrotic subtypes	Capable	Limited
Evaluation of therapy response and post-treatment changes	More informative	Less informative
Depiction of extrahepatic metastatic deposits	More sensitive	Less sensitive
Differentiation between benign and neoplastic lesions (specificity)	Powerful	Limited
Sensitivity	Limited for detecting small lesions	Powerful for detecting small lesions
Absorbed radiation dose	15 - 20 mSV	15 - 20 mSV
Cost	Higher	Lower
Contrast-induced nephropathy risk	Low risk due to limited contrast use in PET/CT	Potential risk due to contrast use in CT

**Figure 3:**
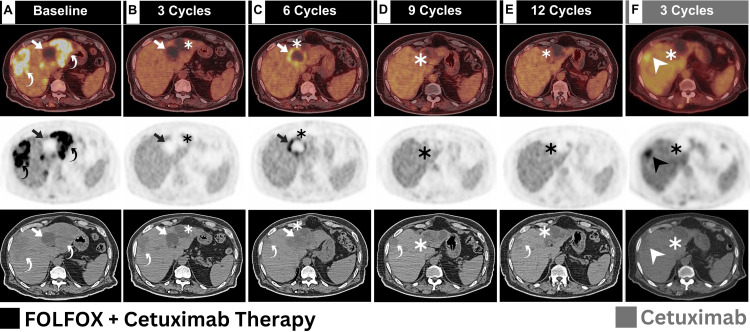
Serial hepatic transaxial images (composed of fused PET/CT, axial PET, and axial CT images for each panel) captured from baseline and following each of the three cycles of therapy received Serial fused [^18^F]FDG PET/CT, PET, and CT liver images were captured from baseline and following each of the three cycles of therapy received. A: At first, several hepatic lesions of various density and metabolic activity were observed (annotations). B: Following the third cycle, all previous hypermetabolic lesions were resolved, yet another hypometabolic lesion (asterisk) emerged near the prominent segment IV necrotic lesion (arrow). C: following the sixth cycle of therapy, the prominent segment IV lesion has established a hypermetabolic rim (arrows), while the segment II lesion has diminished (asterisk). D: After the ninth cycle, morphologic regression of the segment II lesion was witnessed (asterisk), along with a complete response of the aforementioned segment IV lesion. E, F: Additional therapy courses resulted in gradual progression of segment II lesion (asterisks), along with interval establishment of hypermetabolic process in the previously persistent segment VIII hypodensity indicative of viable metastasis (arrowhead). FOLFOX - folinic acid, fluorouracil, oxaliplatin; [^18^F]FDG PET/CT - [^18^F] fluorodeoxyglucose positron emission tomography/computed tomography

In relation to liver metastases, a distinct pattern of response was unveiled. As stated before, the baseline [^18^F]FDG PET/CT depicted multiple metastatic liver lesions of various density and metabolic activity. Several hepatic deposits were noted with only subtle CT hypodensity (Figure 3A, curved arrows).

Whereas, a single segment IV hypodensity with obvious photopenia was depicted, indicative of predominant necrosis (Figure 3A, arrows).­­­ Following the first three cycles, CMR of the previously seen hypermetabolic liver lesions was observed. Despite this, the previously seen necrotic segment IV lesion have maintained the same appearance (Figure 3B, arrows), with establishment of nearby segment II liver lesion (Figure 3B, asterisks). After six cycles of therapy, the prominent segment IV lesion has established peripheral hypermetabolism (Figure 3C, arrows), while the nearby segment II lesion shrunk down (Figure 3C, asterisks). Further shrinkage of segment II liver lesion was observed following the first nine cycles (Figure 3D, asterisks), with CMR of the segment IV lesion. Interestingly, this lesion has established morphologic progression after that (Figure 3E, F; asterisks). Moreover, interval establishment of hypermetabolic process noted involving the persistent hypodensity visualized within segment VIII following the third cycle of cetuximab maintenance, indicative of viable CRLM (Figure 3F, arrowhead). This hypermetabolic lesion have been established at the background of segment VIII liver hypodensity depicted following the third cycle of chemoimmunotherapy and persisted till the twelfth cycle (Figure 3B-E, CT panel; curved arrows). 

**Figure 4:**
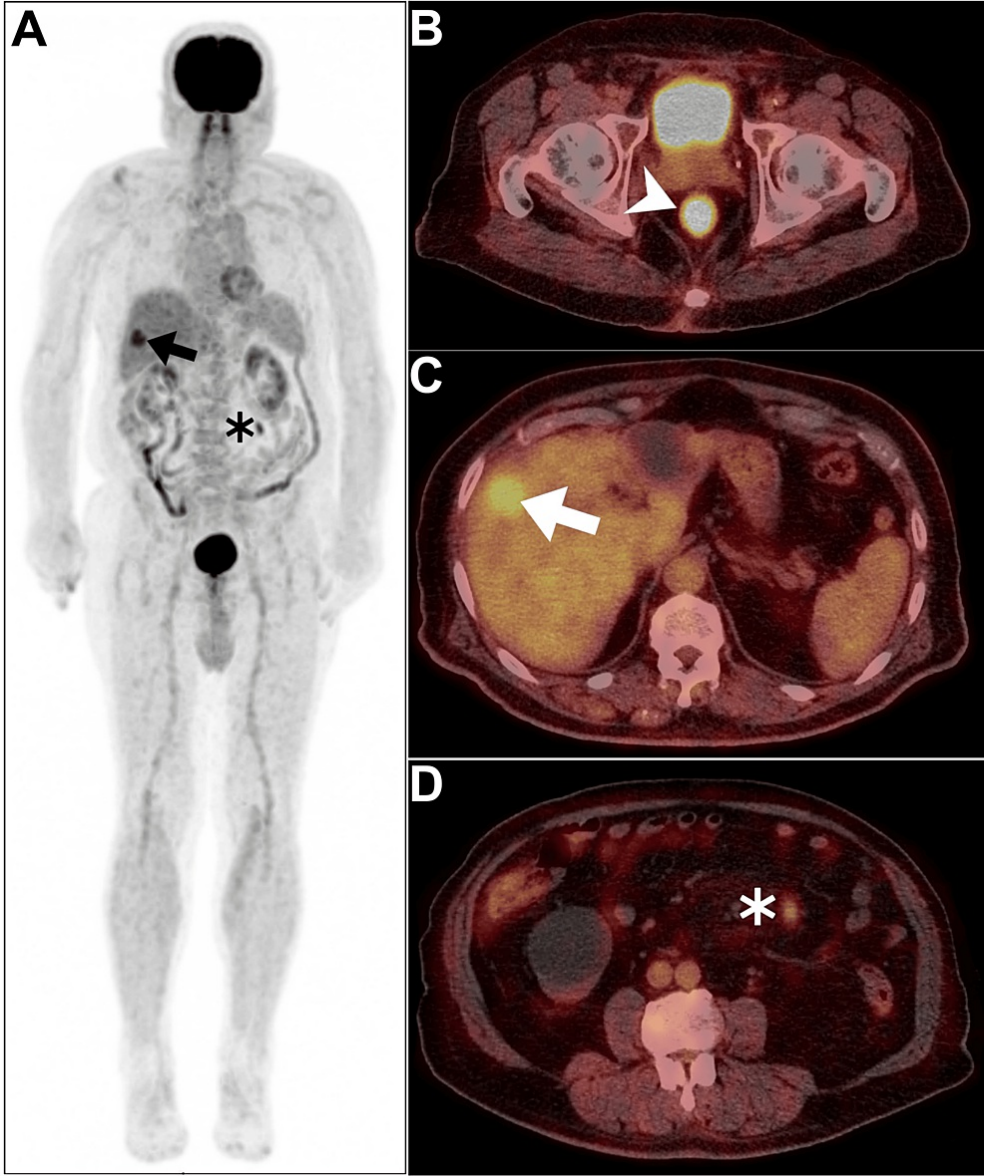
Follow-up PET/CT imaging performed following the sixth cycle of cetuximab maintenance A: Maximum-intensity projection image revealing evidence of two hypermetabolic foci, one of which occupies the liver (arrow), and the other one appears within the peritoneal cavity (asterisk). B: Axial rectal PET/CT image demonstrating more prominent hypermetabolic primary rectal disease (arrowhead). C: Axial hepatic PET/CT image revealing more prominent hypermetabolic hepatic hypodensity indicative of a progressive metastatic liver lesion (arrow). D: Axial abdominal PET/CT image showing interval development of a newly seen hypermetabolic peritoneal deposit (asterisk).

Concerning the patient's long-term follow-up, an extra three cycles of cetuximab were offered, supplementing the initial three cycles, resulting in a cumulative total of approximately six cetuximab-only cycles. Notably, further deterioration was witnessed on subsequent [^18^F]FDG PET/CT imaging following the sixth cycle of cetuximab maintenance (Figure 4).

Therefore, the MDC team is currently contemplating a new round of discussion to determine the most optimal course of action in the immediate future.

## Discussion

Distinguishing between various forms of liver metastases and necrosis is indeed a complex challenge in clinical practice and conventional imaging. Looking back at the serial hepatic [^18^F]FDG PET/CT images, a total of three distinct forms of hepatic hypodensity can be recognized. First, the baseline image depicted hypometabolic segment IV hypodensity, indicative of metastatic lesion undergoing "dirty" or acinar necrosis. Hypometabolic acinar or dirty necrosis has been described and linked to tumor hypoxia caused by a lack of blood supply [11]. Perilesional edema, on the other hand, has been recognized as a cause of metastatic hypometabolism [12].

Secondly, the hypermetabolic segment VIII lesion observed following the third cycle of cetuximab maintenance indicates viable metastatic CRLM [13]. This viable metastatic process superimposed the previous persistent segment VIII subtle hypodensity with a physiologic limit of [^18^F]FDG expression noted before that indicative of indolent metastases [14]. Partial disease response has been observed to result in an augmented CRLM growth of metastases in different animal models. This phenomenon has been associated with the depletion of antiangiogenic factors that are produced by the primary tumor [15]. The equilibrium between proangiogenic and antiangiogenic factors plays a major role in determining whether the metastases will remain in an indolent state or undergo proliferation. Indolent CRLM has been attributed to the release of antiangiogenic factors by the primary tumor, exemplified by angiostatin, which effectively hinders the growth of distant metastases [14]. These indolent lesions typically exhibit subtle hypodensity and are vulnerable to recurrence [14]. The utilization of [^18^F]FDG PET/CT has been employed to identify early recurrence in patients with indolent CRLM subsequent to surgical intervention [14].

In general, necrotic CRLM lesions are typically hypometabolic and hypodense and can hardly achieve satisfactory response [16]. That's why the temporarily responding segment II lesion has established the progression observed in the last two serial [^18^F]FDG PET/CT scans. Interestingly, this lesion has emerged under therapy coverage with an initial partial response followed by paradoxical progression. Occasionally, some necrotic CRLM may exhibit infarct-like necrosis (ILN), which denotes a chemotherapy-mediated healing process [17]. In our case, ILN developed following six cycles of chemoimmunotherapy through the establishment of peripheral hypermetabolism around the previously stable segment IV lesion. This represents the third distinct form of metastatic liver lesion depicted in our patient.

ILN is an uncommon type of necrosis that may manifest in liver metastases originating from colorectal cancer, typically observed after chemotherapy treatment [18]. ILN is frequently associated with lesions that respond well to chemotherapy [19]. Its transient nature, gradually replaced by fibrosis during the healing process, signifies its connection with a positive response to treatment. Efforts have been made to differentiate between the two forms of necrosis through imaging techniques, often involving the analysis of lesion attenuation heterogeneity [20]. ILN may exhibit a more homogeneous appearance on CT scans [2].

The assessment of CRLM response to chemotherapy commonly depends on post-treatment CT scans. Nevertheless, there is a notable absence of adequate knowledge or literature regarding the duration of sustained changes following the completion of treatment. In a considerable proportion of these patients, the treatment effect proves to be temporary, and disease progression swiftly occurs after therapy cessation [10].

## Conclusions

[^18^F]FDG PET/CT holds pivotal importance in evaluating the treatment response of CRLM. Transient necrosis in CRLM appears as a hypodense hypometabolic lesion on [^18^F]FDG PET/CT. However, different types of necrosis may be encountered before and after anti-cancer treatment, with different outcomes. The presence of transient necrosis in CRLM poses a difficulty for imaging modalities. The presented case emphasizes the limitations of relying solely on post-treatment CT scans for assessing CRLM response, highlighting the potential for early rebound recurrence after discontinuing chemotherapy due to partial disease response. [^18^F]FDG PET/CT emerges as a valuable tool for detecting such early recurrences, even in the absence of significant CT changes. This underlines the importance of incorporating functional imaging like PET/CT in the comprehensive management of patients with CRLM. [^18^F]FDG PET/CT can be done during anti-cancer treatment to measure the early response and after the end of anti-cancer treatment to confirm a complete versus partial response and rule out residual or recurrent viable CRLM.
